# Oestrus Analysis of Sows Based on Bionic Boars and Machine Vision Technology

**DOI:** 10.3390/ani11061485

**Published:** 2021-05-21

**Authors:** Kaidong Lei, Chao Zong, Xiaodong Du, Guanghui Teng, Feiqi Feng

**Affiliations:** 1 College of Water Conservancy & Civil Engineering, China Agricultural University, Beijing 100083, China; leikaidong@cau.edu.cn (K.L.); chaozong@cau.edu.cn (C.Z.); fengfeiqi@cau.edu.cn (F.F.); 2 Shandong New Hope Liu he Co., Ltd., Qingdao 266102, China; duxiaodong2@newhope.cn or

**Keywords:** machine vision, bionic boar, sow, oestrus detection, video analysis, welfare

## Abstract

**Simple Summary:**

This study proposes a method and device for the intelligent mobile monitoring of oestrus on a sow farm; this type of monitoring is applied in the field of sow production. A boar model that imitates the sounds, smells, and touch of real boars was built to detect the oestrus in sows after weaning. The models resulted in recognition accuracy rates of 96.12%, 98.25%, and 90.00%. The interaction times and frequencies between the sow and the bionic boar and the static behaviours of both ears during heat were further analysed. The results show that there is a strong correlation between the duration of contact between the oestrus sow and the bionic boar and the static behaviours of both ears. The average contact duration between the sows in oestrus and the bionic boars was 29.7 s/3 min, and the average duration in which the ears of the oestrus sows remained static was 41.3 s/3 min. The interactions between the sow and the bionic boar were used as the basis for judging the sow’s oestrus states. This approach can more accurately obtain the oestrus duration of a sow and provide a scientific reference for the sow’s conception time.

**Abstract:**

This study proposes a method and device for the intelligent mobile monitoring of oestrus on a sow farm, applied in the field of sow production. A bionic boar model that imitates the sounds, smells, and touch of real boars was built to detect the oestrus of sows after weaning. Machine vision technology was used to identify the interactive behaviour between empty sows and bionic boars and to establish deep belief network (DBN), sparse autoencoder (SAE), and support vector machine (SVM) models, and the resulting recognition accuracy rates were 96.12%, 98.25%, and 90.00%, respectively. The interaction times and frequencies between the sow and the bionic boar and the static behaviours of both ears during heat were further analysed. The results show that there is a strong correlation between the duration of contact between the oestrus sow and the bionic boar and the static behaviours of both ears. The average contact duration between the sows in oestrus and the bionic boars was 29.7 s/3 min, and the average duration in which the ears of the oestrus sows remained static was 41.3 s/3 min. The interactions between the sow and the bionic boar were used as the basis for judging the sow’s oestrus states. In contrast with the methods of other studies, the proposed innovative design for recyclable bionic boars can be used to check emotions, and machine vision technology can be used to quickly identify oestrus behaviours. This approach can more accurately obtain the oestrus duration of a sow and provide a scientific reference for a sow’s conception time.

## 1. Introduction

At present, precision livestock farming (PLF) research has attracted the attention of many researchers [1]. The breeding of pigs in a noncontact, stress-free, and healthful manner has always been a research field that scholars worldwide have focused on [2,3,4]. During the process of pig breeding, sows play an important role. By scientifically understanding sows’ oestrus state, one can determine the best mating times of sows and increase the overall embryo implantation rate. The oestrus period is the period in which a sow can accept a boar and achieve ovulation and conception. The oestrus cycle of a sow is composed of four periods: pre-oestrus, oestrus, late oestrus, and dioestrus periods. The average duration of oestrus is 21 days, but there are large individual differences between the phases. The oestrus behaviours of sows are cyclical and transient. When a sow exhibits oestrus behaviour, it needs to be bred in time; otherwise, the breeder will miss the optimal breeding time and must wait for another oestrus cycle. Empty sows lead to increased breeding costs and reduced production efficiency [5,6].

During sow production, oestrus detection is mostly conducted with the manual observation method and boar test method. During the oestrus period, the sow’s feed intake is reduced, its sensitivity to environmental changes increases, and the amount of activity in the pigpen increases [7]. The current general observation method for checking sow emotions is to observe the reaction of the sow to a back pressure test (BPT) and any induced changes in the vulva. A back pressure reaction is one in which sow pigs in oestrus appear to stand still and their ears stand still when their backs are manually pressed on, when they are crawled over by boars, when their backs are hunched, and so on. At the same time, the sow’s vulva becomes red and swollen, and more mucus is excreted from it [8]. The boar test method is used to observe whether a sow has a static reaction through direct contact between a boar and the sow or through the enclosure. This method is simple and convenient to perform but is time-consuming and labour-intensive.

At present, many scholars’ research has shown they have carried out related research on the automatic identification of the process of sow oestrus [9]. Scolari et al. [10] explored the changes in the temperatures of the vulvas and buttocks of sows before and after oestrus; the results showed that the temperature of each sow’s vulva increased significantly at the beginning of oestrus and decreased significantly before ovulation, while the temperature of the buttock surface did not change significantly. Sykes et al. [11] found that the maximum and average temperatures of the vulva during the oestrus period of sows were higher than those during the oestrus cycle period, but there was no difference between the minimum values. Simões et al. [12] found that the temperatures of the sow vulvas first increased and then decreased; the temperatures of the buttock surfaces were not significantly different. Altmann used a small accelerometer to detect sow activity and found that activity during the oestrus period was twice that of a sow that is not in heat [13].

Bressers [14] used accelerometers and set activity thresholds to detect oestrus in sows. The results showed that the acceleration change range of sows during the oestrus period was significantly higher than that of sows during the non-oestrus period. When the threshold was set to 10 m/s2, the amount of exercise detected by the sensor was 10 times higher than that during the non-oestrus period [14]. Freson used infrared sensors to detect the oestrus of sows; the accuracy rate of the proposed method was 86%, the sensitivity of oestrus detection was 79%, and the specificity was 68% [15]. Houwers et al. used sensors to automatically record the frequency of sows visiting boars, and the results showed that when the sows had never been in heat and entered the oestrus period, the frequency of boar visits gradually increased [16]. Korthals counted the average length of sow visits. The statistical results showed that the length of sow visits obeyed the 92 s/d Poisson distribution. The sensitivity of heat detection was 76.4%, and the specificity was 80.3% [17]. Therefore, it is feasible to predict the oestrus time of a sow through the frequency and duration of the sow’s visits to a boar.

With the development of neural networks, more researchers hope to use the autonomous learning capabilities of neural network models to automatically determine the characteristics of recognition behaviour. The advantage of automatically extracting features lies in the robustness and strong adaptability of this approach. In addition, during the learning process, features with higher degrees of discrimination can be automatically distinguished. However, when the resultant model is established, it is necessary not only to pursue accuracy but also to take the running speed of the model into account so that it can be effectively applied in practice.

There has been some development in the recognition of pig behaviour images. Several scholars have studied sow behaviour image recognition, video analysis, and sow behaviour tracking [18,19,20,21]. However, there are few reports on the detection of sow oestrus based on the ‘contact windows’ of machine-vision based bionic boars, and the current research has not been applied to sow oestrus detection and pig production.

Thus, the analysis of the behaviours of sows after weaning based on a ‘contact window’ model of a bionic boar using machine vision technology is herein proposed. This research method is intelligent, offers a non-stressful environment, guarantees sow welfare, and ensures biosafety during the detection process. The main contributions of this article are as follows:

(1) An information model was researched, developed, and built based on the ‘contact window’ of a bionic boar.
(2) The oestrus behaviours of sows were analysed based on machine vision recognition.

## 2. Materials and Methods

To detect the conditions of sows based on machine vision, in this study, a bionic boar detection model was built, which mainly includes an intelligent mobile platform, a bionic model device, an image acquisition device, and a PC processing terminal. Through simulation of the sound and smell of boars, and analysing the response behaviour of sows through machine-vision based imaging technology, a sow oestrus recognition model was established, and the oestrus times of sows can be accurately obtained.

### 2.1. Animals and Housing

To identify the oestrus behaviours of empty-breasted sows, this study was carried out at the pig farms of the Shandong and Chongqing Academy of Animal Husbandry (Rongchang, Chongqing, China) pig farms from July 2019 to December 2020. The test subjects were Yorkshire sows that already been weaned. The sows had 2–3 L. The feeding times were 10:00 a.m. and 4:00 p.m. The test tracked a total of 76 large white sows. The data collection platform included a computer, an intelligent mobile platform, an intelligent detection camera (LRCP106_1080P (Zhi fei, China)), and a bionic model device.

**Figure 1:**
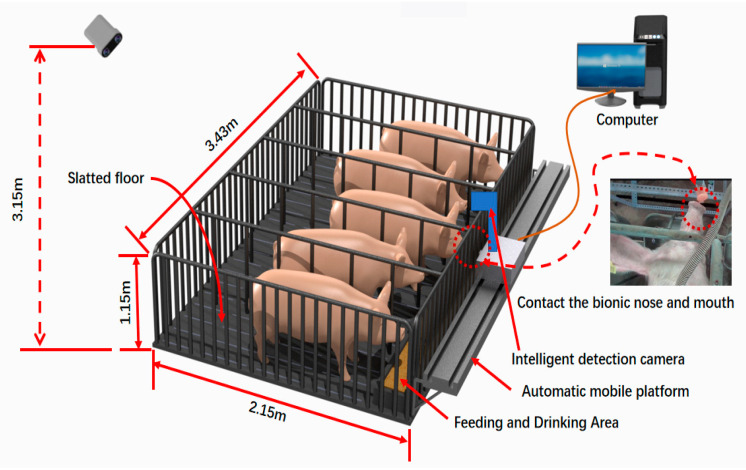
A rendering of the test platform.

In this study, the experimental method mentioned that a total of 76 sows were observed. In other words, 68 sows were observed in the preliminary experiment. At the same time, 68 sows underwent the same experimental observation, the bionic boar, including sound and smell devices that simulate pigs. The actual verification results are based on conducting an inspection with the bionic boar and performing a simultaneous manual back pressing test (BPT) at the same time. A diagram of the test platform is shown in Figure 1.

The intelligent mobile platform used in this article was the FBL80E3500_X linear actuator (FUYU, China), which can be used to set the speed, position, and time through a program compiled in the C programming language. The FBL80E3500_X is a synchronous belt-based linear module. It is a medium-sized closed linear module for synchronous belt transmission. The horizontal speed under a full load is 300–1700 mm/s, the positioning accuracy is 0.1 mm, the horizontal maximum load is 30 kg, and the maximum thrust is 55 N.

For the image acquisition device, the acquisition frequency was set to 30 fps, the resolution was 1280*720, and the collected sow images were transmitted to the PC processing terminal through a data line. A camera was arranged on the upper backside of the circle, and the PC processing terminal was an Intel Core i7 9th generation processor. The bionic model device, a built-in ultrasonic atomization device, was a YuWell (YW, China) NM211C miniature piezoelectric atomizer and it was used to simulate the release of odours from boars; the sound module, the external amplifier of a voice recording playback device, was a Sony ICD-TX650, with a noise reduction function and the advantages of a stereo microphone layout. To increase the authenticity of the bionic model, the front end of the device was designed with a silicone pig nose, and the ultrasonic atomization outlet was a silicone pig nose, which could effectively capture the interactions between a sow and a boar. MATLAB (MathWorks Inc of America.), Origin8.0 (OriginLab Microcal of America), LabVIEW (the Laboratory Virtual Instrument Engineering Workbench of America), and other software were used for image analysis.

### 2.2. Bionic Boar for Verifying the Principle

Studies have shown that the boar test method is often used during production to observe whether the sow’s nose and the boar’s nose will have a standing reaction after touching it. and then to determine whether the sow is in heat. Most chosen boars are middle-aged and old, with strong salivation abilities and docile temperaments. The boars used to provoke the sows should be alternated to avoid having the sows lose interest in a single boar. When arranging the situation, the routes of the boars are planned in advance, the staff are arranged to drive the boars, and fences/gates are set up to block the boars from escaping [22]. At the same time, pressure is put on the back of the examined sow to observe whether the sow has a standing reaction; the knees of the boar are pushed against the sow’s udder, abdomen, and crotch to observe whether the sow is close to exhibiting a standing reaction. It is observed whether changes in the shape and colour of the sow’s vulva occur and whether the sow has frequent urination, irritability, arching, ear erections, body tremors, an inability to eat, and so forth. According to the above procedure for the identification of the sow’s oestrus, it is comprehensively judged whether the sow is in oestrus and the status is marked accordingly [23,24]. Although this process has a high accuracy rate, it is time-consuming and labour-intensive, and there is a certain degree of danger.

It is necessary for boars to be on-site when making love. The boars on pig farms can generally be divided into two categories according to their use: love-making boars and semen-providing boars. Both are essential parts of daily pig farm production. Love-making boars refer to a type of boar used for sex checking, sex induction, and assisted artificial insemination. These boars mainly rely on the smell of saliva to stimulate a sow’s physiological and psychological responses. These ‘odour molecules’ at work are boar pheromones [25].

A boar can stimulate a sow with sight, touch, smell, and sound, the most important of which is smell. Pigs have a large number of olfactory receptors [26]. When a boar appears in front of a sow in heat, the pheromones in the boar’s saliva stimulate the sow’s sexual behaviours, such as standing still, erecting its ears, arching its back, scraping the stall, and so on, within a short time period [27]. After the sow in heat comes into contact with the boar, the pheromone molecules produced by the boar can be sensed through breathing and chewing. This explains why the boar needs to be in contact with the sow’s snout and nose for the processes of sex checking, sex induction, and traditional artificial insemination.

**Figure 2:**
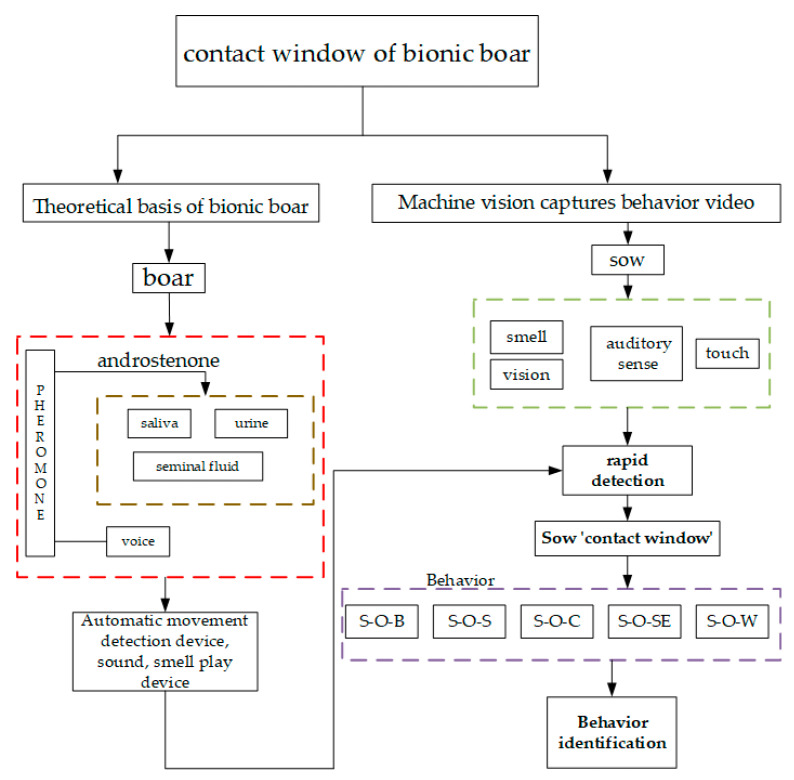
Technical roadmap. Note: S-O-B denotes that a sow in the oestrous cycle bites the rod; S-O-S denotes that the sow stands still during the oestrus cycle; S-O-C denotes that the sow touches the bionic boar snout during the oestrus cycle; S-O-SE denotes that the sow’s ears stand still during the oestrus cycle; S-O-W denotes that the sow moves her head over a wide range during the oestrus cycle.

Building upon the above theoretical basis, we propose a technical route for research on bionic boar-based sow checking, in Figure 2.

Firstly, according to the technical roadmap and the pheromone of the boar to detect the heat of the sow and at the same time to observe the degree of reaction of the sow. Then, by simulating sound and smell of boar and simulating the principle of contact window of boar, a bionic boar was constructed in the experiment. Finally, SOB, SOS, SOC, SO-SE, SOW and other behaviors were analyzed and recognized. Behavior detection can be used as the evaluation of the effect of bionic boars on sows in the estrus cycle. The bionic boar device developed in this study was mainly an intelligent detection device (consisting of a sound-releasing device, an odour-releasing device, an image acquisition device, and a bionic silicone pig nose). A scent device and sound player were installed on the intelligent detection device to check the oestrus of sows. The saliva, urine, semen, and so forth of a boar were contained in the scent release device, and these items were atomized and released through a miniature atomizer. The experimental results show that the combination of the saliva and voices of middle-aged boars leads to long-duration and high-frequency contact with sows. During the investigation, the weaned sow actively touched the detection device, contacted it frequently, had a long contact duration, stood still with both ears motionless, and so forth. These interactions were captured by the camera above the detection device and transmitted to the PC through the end of the accompanying data cable.

During the sow’s bionic boar check-up, the response degree of the sow and the bionic device was observed and recorded. In this study, a bionic boar was used to check up on sows for a period of three minutes per sow. Through the artificial BPT on the sow, observations were made regarding whether both ears of the sow were standing still, whether it had a hunched back, and whether the sow’s pubic area was red and swollen. At the same time, the sow’s response behaviour and frequency were recorded through image technology. The response relationship between the sow and the bionic boar was recorded and studied through video analysis.

### 2.3. Machine Vision Recognition of Oestrus Sow Behaviour in Response to a Bionic Boar

#### 2.3.1. Pre-Processing of Video Image of Bionic Boar

To recognize the response behaviour of a sow in a complex environment, the collected video/image set needed to be pre-processed to reduce background interference and improve the contrast between the target sow and the background. In the home environments of pigs, sows cause imaging problems such as occlusion, complex lighting, and difficulty in capturing target images, and the collection of high-quality images requires computer processing hardware. In MATLAB, the avi2img.m program converted each video into a series of single pictures frame by frame. The grey_frame = rgb2grey(frame) statement was used to convert a colour image into a greyscale image. The contrast between the target sow and the background was improved by transforming the grey level. Establishing different action feature extractions through MATLAB can provide classification data labels for subsequent model classifications.

#### 2.3.2. A Deep Belief Network (DBN) Model for Oestrus Behaviour Identification in Sows

A model is proposed based on bionic boar checking and sow response behaviour identification. Responsive sow behaviours include chewing on railings, standing still, touching the snout of the bionic boar, standing with both ears still, and swinging the head widely. Standing behaviour and contact with the bionic boar snout are the main characteristics that reflect the oestrus behaviour of a sow [3]. By analysing the response behaviour characteristics of sows, a comprehensive evaluation of standing behaviour and combined contact with the snout and nose of the bionic boar is proposed.

##### Establishment of an Oestrus Behaviour Recognition Model for Sows

A deep belief network (DBN) was one of the first non-convolutional models to be successfully applied to deep architecture training. The basic unit of this network is the restricted Boltzmann machine (RBM) [28]. Hinton et al. [29] first used unsupervised pretraining to initialize the network parameters and then adjusted the network parameters through the backpropagation (BP) algorithm. DBNs are widely used in handwriting recognition, image recognition, speech recognition, and other fields [29]. Compared with the traditional multilayer neural network, which is difficult to train, a DBN abstracts high-level features from the observed low-level features by applying a layer-by-layer greedy learning method to realize network training [30,31,32].

DBNs are often trained using the contrastive divergence (CD) algorithm. Assuming that there are d explicit layer neurons and q hidden layer neurons in the network model, let v and h denote the state vectors of the explicit layer and the hidden layer, respectively. In the same layer, each unit satisfies an independent and identical distribution, and the following formulas can be obtained:

(1) $$P\left(v|h\right)=\overset{d}{\underset{i=1}{\prod }}P\left(v_{i}|h\right)$$

(2) $$P\left(h|v\right)=\overset{d}{\underset{j=1}{\prod }}P\left(h_{j}|v\right)$$

In the CD algorithm, the training samples are first assigned to the explicit layer v, and the score of the hidden layer neuron state is calculated according to Equation (2). The probability distribution is determined, and then sampling is performed according to this distribution to obtain h. Then, $v^{\prime }$ is calculated from h according to Equation (1), and $v^{\prime }$ and $h^{\prime }$ are subsequently obtained. The update formula for the connection weight is as follows, where ∆W is the update weight and η is the learning rate.

(3) $$∆W=\eta \left(vh^{T}-v^{\prime }h^{\prime }{}^{T}\right)$$

In image processing, pixels are used as visible layer units to deactivate hidden layer units with a certain probability. By running the divergence algorithm, the network model is trained layer by layer to obtain the weight matrix and offset of the visible unit and the hidden unit of each layer. Finally, according to the weight matrix and offset of each layer, it is judged whether the hidden unit is activated. The distribution of the hidden unit is the abstracted high-level feature.

##### A Sparse Autoencoder (SAE) Model for Female Oestrous Behaviour Recognition

Sparse autoencoder (SAE) neural network technology is an efficient unsupervised feature learning and deep learning classification method. Since it was first proposed, the SAE technology has been widely used in various classification and pattern recognition problems [33]. An SAE neural network classifier includes multiple training layers, and the training output result of the previous training layer is the training input value for the next training layer. The detection process of the SAE neural network classifier described in this section first involves initializing the parameters; then, feedforward conduction is used to train the neural network; finally, a reverse conduction fine-tuning process is used to determine the minimal cost function with the globally optimal parameters, and a collapsed-lane deviation detection classifier is obtained. The SAE neural network integrates all the advantages of a deep neural network and has a strong expression ability.

A stacked autoencoder neural network is composed of multiple layers of sparse autoencoders, and its training process adopts a layer-by-layer greedy training method. The layer-by-layer greedy training method uses the output of the previous layer of an autoencoder as the input of the next layer of the autoencoder to train the network layer by layer in order from front to back. During each step, the first k-11 layers that have been trained are fixed, and then layer k is added; that is, the output of layer k-1 (which has been trained) is the input of layer k to be trained. The feature input set of the stacked autoencoder neural network is called the input layer, the classification result set is called the output layer, and all the layers between the input layer and the output layer are called hidden layers.

Unlike other neural networks, a single-layer SAE is an unsupervised learning algorithm that does not require the training samples to be calibrated. The overall network performance of the single-layer SAE can be obtained via Equation (4):

(4) $$J\left(W,b\right)=\frac{1}{m}\overset{m}{\underset{i=1}{\sum }}J\left(W,b;x^{\left(i\right)},y^{\left(i\right)}\right)+\frac{\lambda }{2}\overset{n-1}{\underset{l=1}{\sum }}\overset{s_{l}}{\underset{i=1}{\sum }}\overset{s_{l}+1}{\underset{j=1}{\sum }}{\left(w_{ij}^{\left(l\right)}\right)}^{2}+\beta \overset{s_{l}}{\underset{j=1}{\sum }}\left(\rho log\frac{\rho }{{\hat{\rho }}_{j}}+\left(1-\rho \right)log\frac{1-\rho }{1-{\hat{\rho }}_{j}}\right)\;$$

Among the parameters, W and b are used to fit the data of the original input $x^{\left(i\right)},y^{\left(i\right)}$, and b is the bias node, also called the intercept. m is the number of feature inputs, and λ is the weight attenuation coefficient, which is used to control the relative importance of the first mean squared error term and the second weight attenuation term to prevent overfitting.$\;w_{ij}^{\left(l\right)}$ is the data weight value from the j-th unit of the l-th layer to the i-th unit of layer l+1. s represents the number of neurons in each hidden layer, β is the weight of the sparsity penalty term, ρ is the introduced sparsity parameter, and ${\hat{\rho }}_{j}$ is the average value of the m input activations of hidden neuron j. $\beta \sum_{j=1}^{s_{l}}\left(\rho log\frac{\rho }{{\hat{\rho }}_{j}}+\left(1-\rho \right)log\frac{1-\rho }{1-{\hat{\rho }}_{j}}\right)$ is the relative entropy between a Bernoulli random variable with a mean of ρ and a Bernoulli random variable with a mean of ${\hat{\rho }}_{j}$ (obtained by measuring the difference between the two distributions)$.\;\frac{\lambda }{2}\sum_{l=1}^{n-1}\sum_{i=1}^{s_{l}}\sum_{j=1}^{s_{l}+1}{\left(w_{ij}^{\left(l\right)}\right)}^{2}$ is a regularization term, and $\frac{1}{m}\sum_{i=1}^{m}J\left(W,b;x^{\left(i\right)},y^{\left(i\right)}\right)$ is the cost function of the model.

##### Support Vector Machine (SVM) Model for Sow Oestrus Identification

The support vector machine (SVM) method is a machine learning classification algorithm based on statistical learning. It is based on the principle of achieving the minimum structural risk, and it was first proposed by Vapnik et al. [34]. Its basic model is a linear classifier with the largest interval in a given feature space. With its solid theoretical foundation and accuracy, the SVM is widely used in text classification and face recognition tasks. In the field of text classification, compared with traditional methods, the SVM approach not only has better robustness but also achieves good results when dealing with high dimensionality [35].

The learning strategy of an SVM is to maximize the category interval, that is, to find the hyperplane with the largest interval in a given feature sample space. This hyperplane separates the two compared types of data and maximizes the geometric interval; this can be approximately understood as a convex quadratic programming problem. The purpose of an SVM is to determine the separation hyperplane that can divide the dataset correctly and obtain the largest set interval. The equation for dividing the hyperplane is shown in Equation (5).

(5) $$W^{T}x+b=0$$

The separation hyperplane is determined by the normal vector $w=\left(w_{1};w_{2};\ldots ;w_{d}\right)$ and the intercept b, which needs to be obtained through training with the sample data. Assuming that the linearly separable training set $T=\left\{\left(x_{i},y_{i}\right)\right\},i=1,2,\ldots ,n,x_{i}$ represents a feature vector in the training set, $y_{i}\in \left\{+1,-1\right\},y_{i}$ is used to input the classification of $x_{i}$ (6):

(6) $$\{\begin{array}{c} W^{T}x+b\geq 1,\;y_{i}=1\\ W^{T}x+b\leq -1,\;y_{i}=-1 \end{array}$$

The distance between a sample point in the space vector and the hyperplane is denoted as D, which can be expressed as follows (7):

(7) $$D=\frac{\left|W^{T}+b\right|}{∥w∥}$$

Among the variables, $∥w∥=\sum_{i=1}^{n}w_{i}^{2}$. The objective function and constraint conditions of an SVM are as follows (8):

(8) $$\{\begin{array}{c} min\frac{1}{2}{∥w∥}^{2}\\ s.t.y_{i}\left(W^{T}x+b\right)\geq 1\left(i=1,2,\ldots ,n\right) \end{array}$$

SVMs are widely used. In addition to text classification, they have also been applied to speech recognition and face recognition tasks, and they have achieved satisfactory results. However, SVMs and other algorithms also have some shortcomings. In cases with small data volumes, the SVM is based on the strategy of minimizing structural risk, and thus, its generalization ability is better. The time required for learning and training in an SVM is longer than for other algorithms.

## 3. Results and Discussions

Regarding weaned sows, mastering detection of the oestrus statuses of sows is a key production process. By checking oestrus with a bionic boar, one can use the degree of response exhibited by a sow as a reference for sow oestrus analysis.

### 3.1. Model Evaluation for Detecting the Oestrus of Sows Based on Machine Vision

**Figure 3:**
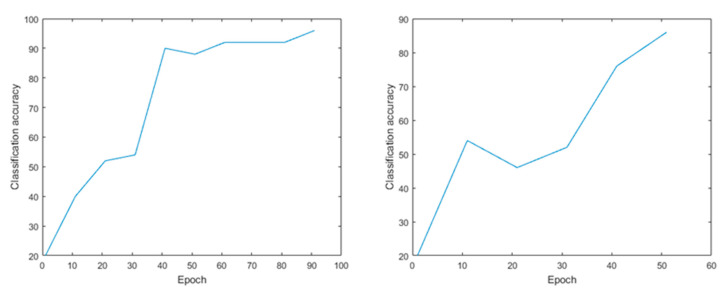
DBN model. Note: The accuracy rate of the method reached 96.12% after 100 iterations and the average error was 3.88%; it was 84.21% after 55 iterations and the average error was 15.79%.

To evaluate the actual effect of recognizing oestrus behaviours in sows based on machine vision detection technology, this article preliminarily verifies the response behaviour of a sow when in oestrus with the bionic boar. We utilized a DBN by applying a layer-by-layer greedy learning method to obtain high-level features from the underlying features and to realize the classification of sow behaviours. The accuracy rate of the proposed method was 96.12%. Figure 3 shows the changes in recognition accuracy after the DBN was applied; the recognition accuracy changed after 55 iterations and 100 iterations.

**Figure 4:**
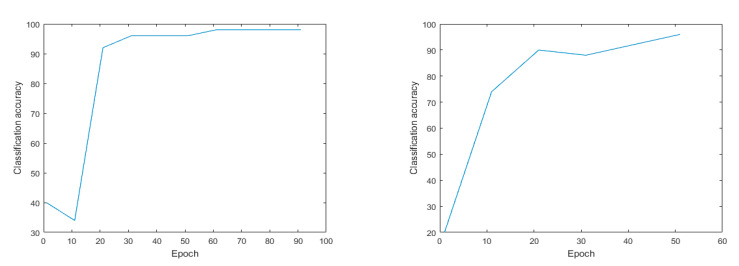
Number of iterations and classification accuracy of SAE model. Note: The accuracy after 100 iterations was 98.25% and the average error was 1.75%; after 55 iterations the accuracy rate was 94.82% and the average error was 5.18%.

The SAE neural network technology is an efficient and unsupervised feature learning and deep learning classification method. The accuracy rate of sow behaviour recognition using this approach reached 98.25%. Figure 4 shows the classification results of the SAE model. After 100 iterations, the accuracy rate reached 98.25%, while after 55 iterations, the accuracy reached 94.82%. Compared with the DBN model, the SAE model has a higher accuracy rate.

**Figure 5:**
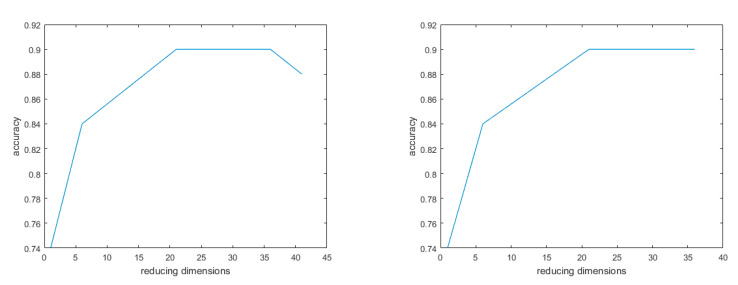
Number of iterations and classification accuracy of SVM model. Note: The accuracy of dimensionality reduction after 45 iterations was 88% and the average error was 22%; after 40 iterations, the accuracy rate was 90.00% and the average error was 10%.

In terms of sow behaviour recognition, after 45 iterations the accuracy of the SVM model was 88%, and the average error was 22%, after 40 iterations, the accuracy rate was 90.00% and the average error was 10%. Compared with the other models, the recognition effect of the SVM model is poor (Figure 5 shows).

Based on the DBN, SAE, and SVM models, we identified the response behaviours of weaned sows, including gnawing on railings, standing still, touching the snout of a bionic boar, standing with both ears motionless, and swinging their heads wildly. The analysis results are shown in Table 1. Note: S-O-B denotes that a sow in the oestrous cycle bit the rod; S-O-S denotes that the sow stood still during the oestrus cycle; S-O-C denotes that the sow touched the bionic boar snout during the oestrus cycle; S-O-SE denotes that the sow had her ears standing still during the oestrus cycle; S-O-W denotes that the sow moved her head over a wide range during the oestrus cycle.

### 3.2. Response of the Sows and Bionic Boars

In this study, the experimental method mentioned that a total of 76 sows were observed. In the preliminary experiment, 68 sows were observed by bionic boars. At the same time, 68 sows underwent the same experimental observation, including a bionic boar sound and smell check). The results show that the bionic boars can achieve the effect of real boars. Finally, 8 sows are randomly selected for analysis and verification. The following focuses on the analysis of 8 sows. The statistical analysis of the test data is divided into two parts. In the first part, the sow data was analysed in a pre-test (68 sows), and the latter part was verified by 8 sows, which can better illustrate the reliability of the method.

#### 3.2.1. Duration of Sow Response

**Figure 6:**
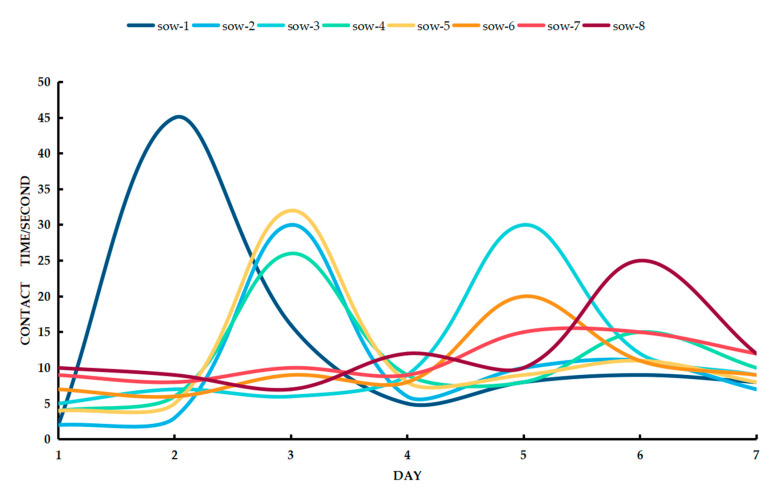
The contact duration between the sow and the bionic boar 1–7 days after weaning (a single sow check lasts three minutes).

The behaviour of a sow is very complicated. Based on video analysis of the response duration of a sow, the physiological condition of the sow, her menstrual cycle, and the influence of the external environment on the sow can be obtained. Some scholars have found that sows usually enter the oestrus cycle within 1–5 days after weaning and are subsequently bred [3]. This article analyses the response durations of sows to bionic boars 1–7 days after weaning. The results show (Figure 6) that there are regular increases and decreases in the duration of the sow’s visit to the bionic boar within those 1–7 days. This is because the average oestrus cycle of a sow is 21 days. During the oestrus cycle, there are regular changes in the hormone levels of sows (luteinizing hormone (LH), follicle-stimulating hormone (FSH), oestrogen, and progesterone levels) [36]. Prolonged contact between a sow and a boar can cause the sow to lose interest [37]. As a result, the data obtained are not accurate. The analysis in this discussion is on the response of the sow to the bionic boar within 3 min.

As shown in Figure 6, Sow 1 frequently contacted the bionic boar device on the second day after weaning, reaching a peak level of contact of 45 s/3 min during the oestrus cycle. The results show that the oestrus of sows is detectable mainly on the third and fifth days. When the bionic boar checked for oestrus and the sow’s oestrus behaviour was detected by video analysis, the average duration of the sow’s contact with the bionic boar was 29.7 s/3 min, and the unit detection time was three minutes or less. Thus, there is a correlation between a sow’s oestrus status and the duration of her response to the bionic boar. To verify the actual effect of the bionic boar, combined with the daily inspection of the sows, the back of each sow was manually pressed. The indicator of oestrus was the sow’s response to back pressure (i.e., the BPT) [38]. Within 1–7 days after weaning, when the sow was not in heat, the average duration of contact with the bionic boar was 8.44 s/3 min. On the second day after weaning Sow 1, the sow’s response time reached 45 s/3 min. At the same time, the manual BPT was performed, and the test results are consistent; all the sows were in oestrus.

The response durations of weaned sows to the bionic boar were analysed through video analysis. The results show that when a sow was not in heat, the sow was curious about the bionic boar and interested in the ‘pheromone’ (saliva, urine, semen, etc.) released by the bionic boar and the recorded sound of the boar. During this period, the average response duration of weaned sows to the bionic boar was 8.44 s/3 min. However, an excessively long response time was recorded on a certain day during this cycle. When the weaned sow had a higher contact duration on one day than on other days, At the same time, the results of artificial back pressure detection of estrus on sows are often in estrus.

#### 3.2.2. Duration of Response (Frequency)

The target of experimental data analysis, from the first day of weaning to the sow’s artificial back pressure detection, was the cut-off of oestrus. The number of responses (frequency) of the weaned sow to the bionic boar was also an indicator of the physiological condition of the sow. The bionic boar model proposed in this paper has real boar smell, sound, and touch devices. The video analysis results show that the number of times that the sow is in contact with the bionic boar after weaning changes regularly with the number of days of post-weaning.

**Figure 7:**
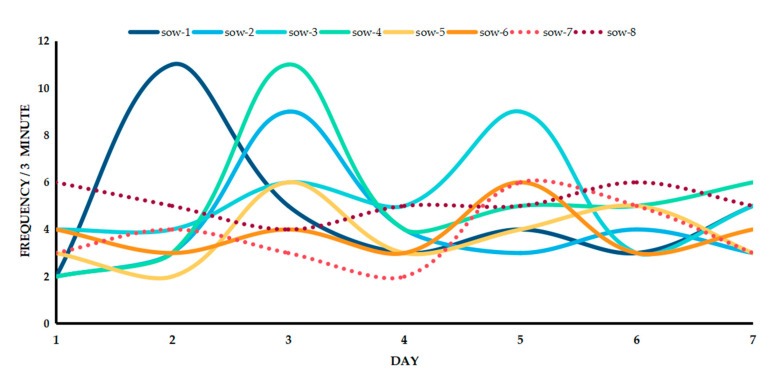
The number of times the sow was in contact with the bionic boar 1–7 days after weaning (within three minutes of a single love check).

When the sow was detected as being in oestrus under artificial back pressure, the weaned sow frequently came into contact with the bionic boar device. In the unit’s 3-min investigation time, the average number of interactions with the bionic boar was 8 times/3 min. When not in heat, the average number of contacts was 3.8 times/3 min. The data show that when a sow was detected as exhibiting oestrus under artificial back pressure, a crest curve appeared during the oestrus cycle. When the weaned sows were in heat, the average number of exposures to the bionic boars was 8.3 times/3 min. In Figure 7, Sow 7 and Sow 8 are represented by the dotted lines. Through video analysis, although Sow 7 exhibited curve fluctuations, there was no oestrus behaviour detected upon the application of manual back pressure. The curve in the figure corresponding to Sow 6 shows a regular rise and fall. The result of manual detection is that the oestrus state was not obvious. Finally, mating was carried out, and the tracking results show that Sow 6 returns to oestrus after mating, but the mating was unsuccessful.

According to the verification data obtained from the pig farm, the number of sow responses to the bionic boar was used to determine whether the sow was in oestrus, and the accuracy rate was 87.5%. Research has shown that in the field, radiofrequency identification (RFID) and other sensors are used to monitor the frequency of sow visits to boars to detect oestrus [39,40,41]. The result was compared with that of the artificial BPT, and the preliminary statistics in the field test exceeded 60%. It is concluded that compared with manual observation, subjective observation can be quantified into image analysis, which is different from previous studies. The subjective observation of previous studies is time-consuming and laborious. However, real boars are used for checking in this situation. In contrast, this study used a bionic boar device for checking, which offers time savings, labour savings, and automated operation. The most important aspect is that the bionic boar device is a recyclable and sustainable device.

#### 3.2.3. Duration of the Static Response of Both Sow Ears

The experimental object in this study was a large white sow that had good reproductive performance and a high number of surviving piglets. During oestrus, large white sows often exhibit some signs that they are actually in oestrus. With a decrease in feed intake and an increase in activity, a sow’s vulva appears red and swollen, the sow’s limbs often appear to stand still, and the ears are upright.

**Figure 8:**
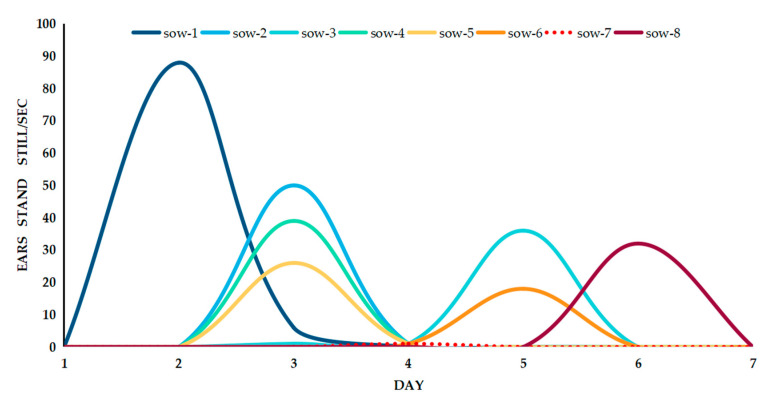
The time until a sow’s ears stand still 1–7 days after weaning (within three minutes for a single oestrus check).

In a pig house, the staff is required to have experience and theoretical knowledge to be able to judge whether a sow is exhibiting oestrus behaviour based on physical signs. We used time quantification to determine the duration of a sow’s body movements. As shown in Figure 8, the response of the ears of a weaned sow (erect) to the bionic boar was more obvious during the oestrus period than in other periods. In Figure 8, the peak of the binaural standing occurred during oestrus, and the duration of binaural standing was higher when the sow was in oestrus than when it was in the non-oestrus state. On the second day after weaning, Sow 1 had both ears upright for 88 s/3 min when the bionic boar performed an oestrus check, with each check carried out once every 3 min. Combined with the artificial BPT, the results all show that the sows were in oestrus. According to the data analysis in Figure 8, the average standing time of both ears in the sows in heat was 41.3 s within a single 3-min check. When a sow is not in oestrus, its ears will not stand still for a long time. According to the response behaviour of the sows and the bionic boars during oestrus in the early stage, a preliminary statistical analysis was carried out on the 8 sows in the field verification test. The later tracking results showed that Sow 6 returned to oestrus after unsuccessful breeding. The rest of the verified sow samples all entered the gestation stage. A threshold regarding the length of time that the ears of a sow are erect can be used as the basis for the judgement of oestrus states.

### 3.3. Evaluation Rule of the Response of a Yorkshire Sow to a Bionic Boar from Weaning to Oestrus

During the period from weaning to oestrus, the behaviour changes of a sow can be determined through video analysis technology. The SAE model can be used to classify and recognize sow behaviour, enabling one to achieve mastery of a sow’s oestrus status. For the research in this article, an analysis and a comparison of multiple neural network-based classification and recognition models with respect to sow behaviours during the oestrus cycle were performed. The maximum accuracy rate reached 98.25% (minimum 90.00%), and the detection effects of these models are superior to that of the method of Freson [15] (86%), which uses infrared sensors to detect sow oestrus. At the same time, the use of bionic boars combined with machine vision technology for oestrus detection shows intelligence and mindfulness of sow welfare. This is an obvious advantage over detection with traditional sensors [16]. Through binaural erection analysis of the response degree of a weaned sow to the bionic boar, including the duration of the response to the bionic boar device, the number of visits, and the actual response of the sow, a comprehensive evaluation of the oestrus status of the sow can be achieved with a high accuracy rate.

**Figure 9:**
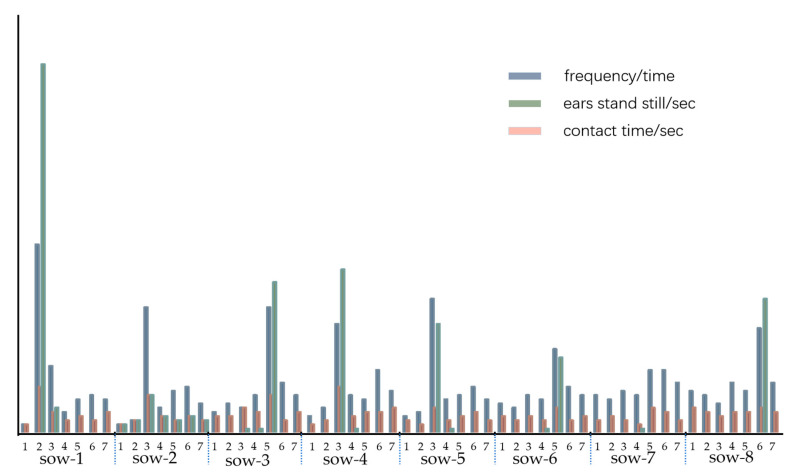
In Figure 9, data columns of different colours on the Y axis represent different units, so they are placed under the same Y axis in order to visually display the data distribution. The response of a sow to the bionic boar (frequency, duration of ears standing still, duration of body standing still). Frequency/time is the number of instances of contact, that is, the frequency of contact per unit time; ears stand still/sec is the static time of both ears of the sow in response to the bionic boar in seconds; contact time/sec is the duration of contact between the sow and the bionic boar in seconds.

As shown in Figure 9, when the bionic boar was checking the oestrus status of a sow, the frequency of the sow’s response (standing still with both ears up) and the frequency of contact with the bionic boar’s silicone snout device were strong correlation factors related to the sow’s oestrus behaviour. However, the duration of the exposure of the sow to the biomimetic boar snout device on its own was a relatively weak factor. The possible cause for this is that the bionic boar did not induce enough sexual attraction in the sow.

Figure 9 shows the actual verification results of conducting an inspection with the bionic boar and a simultaneous manual BPT at the same time. For the sow and the bionic boar device, if the duration of contact and the time that both ears stand still were significantly higher than the same metrics in the previous test, the sow was judged to be in oestrus (quantify the results of manual observation through the method of data analysis and display). The manual pressure back test was performed simultaneously, and the test results are found to be consistent. Through the continuous tracking of breeding, it was found that Sow 6 returned to oestrus and that breeding was unsuccessful. Sow 7 was determined to have not been in heat. The reason for this is that the body condition of a pig is poor after weaning, and the skeleton of the sow’s back can be clearly seen. Poor body conditions or high obesity can directly affect a sow’s oestrus status [42]. This method is aimed at a single breed, Yorkshire sows, and three-parity sows with regular oestrus periods were selected. In the future, the method can be extended to the oestrus monitoring of gilts.

## 4. Conclusions

In this study, a bionic boar model was built to check the conditions of weaned sows in response to the saliva, urine, and semen of a boar. Furthermore, based on video analysis technology, a neural network model was used to classify the behaviours of large white sows during the oestrus cycle. The results show that the recognition accuracy of the SAE model was 98.25%. It can effectively identify the oestrus behaviours of sows by analysing the response degree of each sow to the bionic boar. The results show that there was a strong correlation between the contact duration of the oestrus sow with the bionic boar and the static behaviours of both ears during the oestrus cycle. The average duration of contact between the sows in oestrus and the bionic boar was 29.7 s/3 min, and the average length of time that the ears of the oestrus sows were upright was 41.3 s/3 min. The interaction between a sow and the bionic boar can be used as the basis for judging the sow’s oestrus state.

## Figures and Tables

**Table 1:** Recognition accuracy rate of three models for five behaviours. The average error was 5.17%.

Model	DBN	SAE Accuracy (%)	SVM
Behaviour			
S-O-B	90.00	80.00	90.00
S-O-S	100.00	99.94	90.00
S-O-C	60.86	80.21	80.00
S-O-SE	100.00	100.00	100.00
S-O-W	80.00	80.00	80.00

## Data Availability

All data sets during the current study are available from the corresponding author on fair request.
